# Experimental hybridization and backcrossing reveal forces of reproductive isolation in *Microbotryum*

**DOI:** 10.1186/1471-2148-13-224

**Published:** 2013-10-10

**Authors:** Britta Büker, Elsa Petit, Dominik Begerow, Michael E Hood

**Affiliations:** 1 Lehrstuhl für Evolution und Biodiversität der Pflanzen, AG Geobotanik, Ruhr-Universität Bochum, Universitätsstraße 150, Bochum 44780, Germany; 2 Department of Biology, Amherst College, 220 South Pleasant Street, Amherst, MA 01002, USA

## Abstract

**Background:**

Hybridization and reproductive isolation are central to the origin and maintenance of species, and especially for sympatric species, gene flow is often inhibited through barriers that depend upon mating compatibility factors. The anther-smut fungi (genus *Microbotryum*) serve as models for speciation in the face of sympatry, and previous studies have tested for but not detected assortative mating. In addition, post-mating barriers are indicated by reduced fitness of hybrids, but sources of those barriers (i.e. ecological maladaptation or genetic incompatibilities) have not yet been detected. Here, backcrossing experiments, specifically controlling for the fungal species origins of the mating compatibility factors, were used to investigate reproductive isolation in the recently-derived species *Microbotryum lychnidis-dioicae* and *Microbotryum silenes-dioicae*.

**Results:**

Assortative mating was detected during backcrossing and was manifested by the preferential conjugation of the hybrid-produced gametes with non-hybrid gametes containing mating compatibility factors from the same parental species. Patterns of post-mating performance supported either a level of extrinsic isolation mechanism, where backcross progeny with a higher proportion of the pathogen genome adapted to the particular host environment were favored, or an infection advantage attributed to greater genetic contribution to the hybrid from the *M. lychnidis-dioicae* genome.

**Conclusion:**

The use of controlled backcrossing experiments reveals significant species-specific mating type effects on conjugations between recently-derived sister species, which are likely to play important roles in both maintaining species separation and the nature of hybrids lineages that emerge in sympatry between *Microbotryum* species.

## Background

Mating between sympatric species is a regular occurrence in natural populations, and increasing anthropogenic re-distributions of organisms is driving ever greater frequencies of species contact and hybridization [1,2]. Whether giving rise to new, independent hybrid lineages or to the movement of alleles between species (i.e. introgression), recent population genetics and experimental studies show that inter-specific mating can be a major force behind adaptation and speciation [3-5]. The potential for hybridization to contribute to biodiversity, however, involves the interactions of multiple processes that remain incompletely understood [6]. In particular, hybridization is limited by a complex interplay of pre- and post-mating reproductive barriers that can decrease mating compatibility between species or the fitness of hybrid individuals [2].

Especially in sympatric species, isolating mechanisms that depend on mating behaviors (i.e. compatibilities of different sexes or mating types) play a central role in reproductive isolation. For animals and plants, it has been shown that mating (mate interaction) and gametic (gamete interaction) behavior are effective mechanisms to keep species separated [7]. In fungi the influence of mating patterns is less transparent, partly because of the complexity and variety of fungal mating systems [8]. Many fungi persist as haploid individuals, where compatibility is determined by molecular signals, rather than a reliance on genotypically determined anisogamy. In most fungi, mating depends on the compatibility of pheromones and pheromone receptors, and thus, those proteins and their genes might play a central role for reproductive isolation. Although studies show the importance of assortative mating, and even reinforcement for reproductive isolation in fungi, evidence that these are due to the pheromone and pheromone receptor specifically is indirect [9-11].

Genetics of the mating compatibility system might also be effective in the post-mating stage and thus influence hybrid’s fitness potential. For instance, in mating systems, where homogametic and heterogametic sexes occur (e.g. XY or ZW systems in mammals or birds, respectively), hybrid inviability occurs more often in the heterogametic sex than in the homogametic sex [12], which often attributed to the hemizygosity nature of the structurally divergent sex chromosomes [13-15]. In fungi, where structural heterozygosity of sex chromosomes also occurs, asymmetrical effects upon hybrid fitness depending on the particular combination of sex chromosomes has also been observed [16] - in analogy to Darwin's corollary to hybrid viability [17].

In the present study we utilize members of the basidiomycete fungi in the genus *Microbotryum* to analyze the effect of the mating type on reproductive isolation. *Microbotryum* comprises many fungal species that typically specialize to a given host plant species [18-22]. The sibling species *M. lychnidis-dioicae* and *M. silenes-dioicae* (hereafter referred to as M-Sl and M-Sd) infecting *Silene latifolia* and *Silene dioica*, respectively, can hybridize in natural overlapping habitats, but frequency of hybrids is low [23,24]. As typical for fungi, mating in *Microbotryum* occurs during the haploid stage and is controlled by a special region in the genome (MAT), which is responsible for the production of pheromones and pheromone receptors. In *Microbotryum*, the MAT region is located on a pair of non-recombining and size-dimorphic mating type chromosomes [25]. The different mating types are referred to as a1 and a2 and haploid conjugation occurs exclusively between cells of opposite form [26].

The role of the mating system and mating type during reproductive isolation, and especially effects that are linked to the MAT region, remain unclear in *Microbotryum*. Generally, most *Microbotryum* species have high selfing rates [24], thus limiting the probability of interspecific gene flow [26-28]. Mechanisms of pre-mating barriers in the form of assortative mating have been investigated but not found [29,30]. When hybridization is achieved experimentally, it often leads to the production of unbalanced meiotic products with limited growth and reduced infection ability [30]. This loss of F1-hybrid’s fitness in *Microbotryum* seems to increase with the genetic distance among crossed species [31,32]. In addition, maladaptation to the extrinsic host environment seems to be important in *Microbotryum*, where hybrids are less successful in producing complete infection symptoms than non-hybrids on the parental host environment [31].

Here, we aim to analyze determinants of mating type effects on reproductive isolation between the recently-derived *Microbotryum* species *M. lychnidis-dioicae* and *Microbotryum silenes-dioicae*[24]. This is achieved by backcrossing experiments that can manipulate the identity between paired mating partners at the MAT regions and the rest of the genome. First, we test whether assortative mating occurs with regard to the mating type locus in the hybrid-produced gametes backcrossing combinations with gametes from parental species. Secondly, we quantify the fitness of backcrosses of F1-hybrids on different host environments to assess the contribution of mating type effects and extrinsic factors.

## Results

### Evidence for assortative mating in F1-hybrids

Conjugation rates of 25 haploid isolates of F1-hybrid gametes between *Microbotryum lychnidis-dioicae* and *M. silenes-dioicae* backcrossed to parental, non-hybrids isolates provided evidence of assortative mating depending on the species’ specific MAT region. By using meiotic products from F1-hybrids to backcross with parental (non-hybrid) gametes, the paired alleles at the mating type locus in particular were manipulated to be from the same species (homospecific backcross) or the alternate species (heterospecific backcross) for the comparison of conjugation rates. No mating type-associated or species-associated differences for conjugation proportions could be detected (mating type: t (23) = - 1.12, p = 0.281; gamete origin: t (23) = - 0.23, p = 0.823), and therefore isolates were pooled in the test for difference in homospecific versus heterospecific backcrossing rates. Conjugation rates for backcrossing were significantly greater where the mating type alleles of hybrid- and parental-derived sporidia were from the same *Microbotryum* species (i.e. homospecific backcrossing) than when backcrossing combined mating type alleles were derived from different *Microbotryum* species (i.e. heterospecific backcrossing) (paired t-test: t (23) = 3.84, p = 0.001) (Table 1).

**Table 1:** Contrast of mating proportions of homospecific versus heterospecific backcrosses

	**N**	**M**	**SE**	**MED**	**p**
T-Test*					0.001
*Homospecific*	24	0.38	0.21	0.32	
*Heterospecific*	24	0.27	0.15	0.20	

*Two-tailed paired T-test; N: number of tested isolates; M: mean; SE: standard Error; MED: median; p: significance level (α = 0.05).

**Figure 1:**
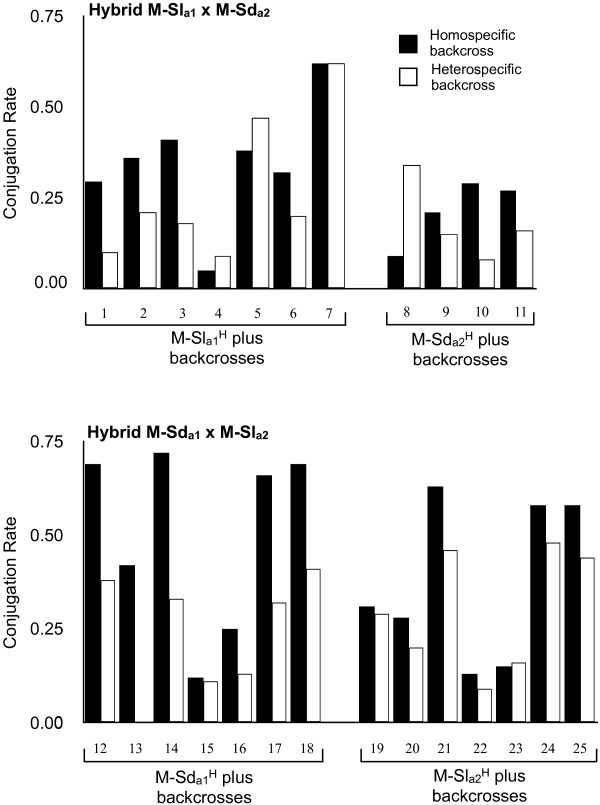
**Conjugation rates of experimentally-produced *Microbotryum *F1-hybrids with their backcrosses.** Upper diagram shows observed conjugation rates for haploid isolates resulting from F1-hybrid type M-Sl_a1_ × M-Sd_a2_ and lower diagram from M-Sd_a1_ × M-Sl_a2_. All 25 haploid hybrid isolates have been backcrossed to parental isolates harboring the same species´ mating type (homospecific backcross) and to parental gametes harboring the different species´ mating type (heterospecific backcross).

Successful mating was observed for 24 of 25 combinations. The exception was isolate #13, which conjugated with the parental isolate in the homospecific backcross but not in the heterospecific backcross (Figure 1). The control for this isolate conjugated with the haploid of opposite mating type derived from the same F1-hybrid (Additional file 1, C6). Because the reason of failure of isolate #13 to conjugate in the heterospecific backcross remains unclear (i.e. experimental noise or strong assortative mating), isolate #13 has been excluded from statistical analysis. The number of cells counted per conjugation test varied from 82 to 610 among the paired crosses, and the proportion of cells involved in conjugations after 12 hours of incubation averaged 0.32 (SE = 0.03) with considerable variation among paired mixtures (Figure 1, Additional file 1).

### Post-mating experiment reveals some significant differences among treatments but the patterns did not point to a single explanatory mechanism

**Figure 2:**
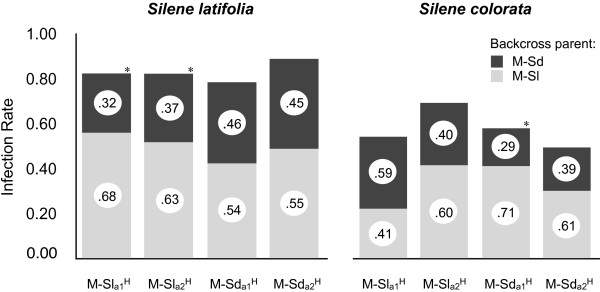
**Infection rates and proportion of backcross genotypes for each given F1-hybrid genotype.** F1-hybrid isolates were backcrossed to parental isolates and inoculated to the hosts *S. latifolia* and *S. colorata.* Infection rate is indicated by the height of the bars, while number in the circle shows the proportion of backcross genotypes of diseased plants.

With a backcrossing design that manipulated the host environment, the proportion of the F2-hybrid pathogen genome derived from M-Sl or M-Sd, and the homospecificity/heterospecity of the mating type locus, extrinsic and intrinsic factors contributing to post-mating reproductive isolation were investigated. All *Microbotryum* genotypes produced by hybrid backcrossing caused anther-smut disease following inoculation of the natural host *S. latifolia* and the artificial host *S. colorata*[33], allowing assessment of the influence of native versus novel host environment (extrinsic factors) and mating type region (intrinsic factors). In total, 585 *S. latifolia* plants flowered and the mean infection rate across different gamete types were 0.83 (SE = 0.07), while on *S. colorata* the flowering individuals (414 in total) were diseased at the rate of 0.59 (SE = 0.05) (Table 2, Figure 2).

**Table 2:** Infection rates and proportion of homospecific backcrosses for the four given hybrid genotypes

Gamete type* and isolates no.	***Silene latifolia***					***Silene colorata***				
	**A**	**B**	**C**	**D**	**p**	**A**	**B**	**C**	**D**	**p**
**M-Sl**_**a1**_^**H**^	**147**	**123**	**120**	**0.69**	0.00	**116**	**63**	**37**	**0.39**	0.32
1	37	32	31	0.52		29	13	4	0.50	
2	33	28	28	0.89		33	17	12	0.58	
3	44	35	32	0.72		28	15	13	0.46	
4	33	28	29	0.62		26	18	8	0.00	
**M-Sl**_**a2**_^**H**^	**153**	**126**	**100**	**0.63**	0.01	**98**	**68**	**63**	**0.59**	0.13
19	42	37	23	0.52		28	21	18	0.61	
20	37	32	24	0.58		29	22	19	0.58	
22	39	30	29	0.69		21	14	16	0.75	
23	35	27	24	0.71		20	11	10	0.40	
**M-Sd**_**a1**_^**H**^	**140**	**110**	**52**	**0.46**	0.68	**93**	**54**	**42**	**0.33**	0.02
12	41	36	15	0.67		22	12	7	0.43	
13	30	23	12	0.25		23	14	10	0.50	
15	37	29	17	0.41		28	15	14	0.21	
16	32	22	8	0.50		20	13	11	0.18	
**M-Sd**_**a2**_^**H**^	**145**	**129**	**67**	**0.45**	0.46	**107**	**53**	**41**	**0.41**	0.21
8	34	30	18	0.39		19	8	7	0.43	
9	33	30	15	0.53		24	14	11	0.27	
10	37	33	18	0.61		34	13	14	0.36	
11	41	36	16	0.25		41	18	9	0.56	

*M-Sl_a1_^H^: haploid hybrid genotype with a1 mating type chromosome originating from *M. lychnidis-dioicae;* M-Sd_a2_^H^: haploid hybrid genotype with a2 mating type chromosome originating from *M. silenes-dioicae (*and vice versa for M-Sd_a1_^H^ and M-Sl_a2_^H^). A: number of plants inoculated; B: number of infected plants; C: number of infected flowers genotyped; D: proportion of infection arising from homospecific backcrosses, p: binomial probability. Numbers in bold: summed (A,B,C) and averaged (D) values for the four isolates of each gamete type.

On the natural host *S. latifolia*, hybrid isolates where the mating type chromosome originated from *M. lychnidis-dioicae* (M-Sl^H^) that were backcrossed with parental, non-hybrid M-Sl (i.e. homospecific backcross for mating type) exhibited superior competitive infection ability than M-Sl^H^ heterospecific backcross to M-Sd (Figure 2) (2-tailed Binomial-test, M-Sl_a1_^H^: N = 120, p = 0.00; M-Sl_a2_^H^: N = 100, p = 0.01). For hybrid isolates where the mating type chromosome originated from *M. silenes-dioicae* (M-Sd^H^), the more frequent infection was the backcross to the mating-type heterospecific M-Sl rather than to the homospecific M-Sd, but the differences were not statistically significant (2-tailed Binomial-test, M-Sd_a1_^H^: N = 52, p = 0.68; M-Sd_a2_^H^: N = 67, p = 0.46) (Table 2).

On the non-host species *S. colorata*, intended to serve as an environment to which neither M-Sl nor M-Sd were better adapted, M-Sl^H^ haploid genotypes did not show a statistical bias in the backcross direction (homospecific versus heterospecific) among infected plants (2-tailed Binomial-test, M-Sl_a1_^H^: N = 37, p = 0.32; M-Sl_a2_^H^: N = 63, p = 0.13). However for M-Sd^H^ haploids, the M-Sd_a1_^H^ significantly deviated from the 0.5 ratio with most infections being from the heterospecific backcrossed type (2-tailed Binomial-test, N = 42, p = 0.02). M-Sd_a2_^H^ did not show a statistical bias in the backcross direction (homospecific versus heterospecific) among infected plants (2-tailed Binomial-test, N = 41, p = 0.21) (Table 2).

## Discussion

The importance of mating between species in nature is becoming more apparent as molecular studies reveal extensive evidence of hybridization events. Thus, the study of hybrids and reproductive isolation is now central to our understanding of the origin and maintenance of species [4,6,34]. In the present study, the use of experimental hybrids between the anther smut fungi, *M. lychnidis-dioicae* and *M. silenes-dioicae*, helps to illuminate the effect of mating type during reproductive isolation in recently-diverged sister species. The results show significant support for pre-mating barriers depended on the combinations of specific mating type chromosomes and less obvious signs for post-mating isolation driven by the origins of the mating types. In contrast to previous hybrid studies in this system, the use of controlled backcross experiments further reveals effects which are likely to play important roles in both maintaining species separation and the nature of backcrossed hybrids lineages that may emerge in the presence of backcrossing potential.

### Assortative mating in *Microbotryum*

Pre-mating barriers between sister species, which may contribute directly to their evolutionary viability and isolation, is an issue complicated by the multiple influences upon contact and mating between individuals. In the anther smut fungi that infect the Caryophyllaceae, where the pathogens are specialized to their particular hosts [18,19,22,30], the strength of pre-mating barriers in sympatry is poorly understood. Sympatric populations of *M. lychnidis-dioicae* and *M. silenes-dioicae* are common but the frequency of hybrid genotypes seems to be low [23,24]. Previous studies have generally not detected causes for pre-mating isolation upon contact between species [30], apart from the potential contribution of a developmentally influenced high selfing rate in combination with sibling competition [28]. The current study shows that assortative mating, in the form of recognizing species-specific variation at the mating type locus, might serve as pre-mating barrier that is active between sister species of *Microbotryum*. Although the signal is significant, patterns for assortative mating among the two species are weak. This might be due to the fact that the most closely related *Microbotryum* species have been used - out of the necessity to have viable F1-hybrid meiotic products, and we may expect stronger MAT- predicted assortative mating in more distant other species pairs that come into contact in nature. Moreover, the large variation in conjugation rates among replicates emphasizes that mating in *Microbotryum* might be also influenced by other factors that have not been isolated yet.

For example, in the study by Le Gac et al. [30], assortative mating was evaluated by testing for the correlation between mating rates (intra- and interspecific) and the genetic distances between several *Microbotryum* species. Wide variation in mating rates was seen across the species pairs, but it was not correlated with genetic distances. That result also suggests that species differences besides the compatible mating types may be affecting conjugation rates (i.e. environmental responses, phenology, etc.). In *M. lychnidis-dioicae*, previous studies have shown that temperature, available nutrients, and the presence of the plant exudate alpha-tocopherol can affect the propensity of haploid cells to mate [35-37], as other organisms may respond similarly to extrinsic signals such as pH or light [38,39]. In the current study, gametes were produced from F1-hybrids, where the identity of the non-recombining mating type chromosome was controlled for and the autosomal component of the genome is generally expected to be a mixture of the two parental species, thus potentially homogenizing the influence of contrasting cellular responses to non-pheromone-based environmental cues. Therefore, with this approach the influence of mating types upon behavior could probably be better resolved than in prior studies to reveal preferences in the mating compatibility signals.

With evidence that *M. lychnidis-dioicae* and *M. silenes-dioicae* originally diverged through allopatric isolation [24], we see now that the pathogens show adaptation to specific hosts that could further contribute to their isolation [40]. Neutral divergence or selection for assortative mating upon secondary contact (i.e. reinforcement, but see [41]), remain plausible explanations for the evolution of the patterns observed here.

### Sources of post-mating isolation

Fitness reductions due to mal-adaptation to parental environments and genomic-level incompatibilities that are typical of hybrids have been experimentally demonstrated using crosses between *Microbotryum* species. Interspecific *Microbotryum* hybrids are less successful at infecting host plants than the progeny of intraspecific crosses [30]. Also, hybrids often show incomplete sporulation on host plants [31,32]. Moreover, the study of Le Gac et al. [30] revealed the existence of host-dependent factors that influence hybrids fitness, where identical F1-hybrid genotypes between *M. lychnidis-dioicae* and *M. silenes-dioicae* differed in infection ability on their two hosts, *S. latifolia* and *S. dioica*. Our results may support the conclusion of host-dependent effects upon hybrid fitness, where backcrossing that was homospecific for the *M. lychnidis-dioicae* mating type was significantly favored on *S. latifolia* but not on *S. colorata*, consistent with the expectation that host adaptation to *S. latifolia* is an extrinsic post-mating factor [42]. This coincides with the meta-analysis study of Giraud and Gourbier [43] that also emphasizes that the occurrence of post-mating barriers in *Microbotryum* is more likely caused by extrinsic factors than genetic incompatibilities.

In our design, the second natural parental host environment could not be used, (i.e. *Silene dioica*), but it would be very informative to test forces of extrinsic isolation in models that can include both parental hosts as environments. The use of a novel host environment did, however, allow our study to assess mating compatibility based upon the species-specific mating type chromosomes. In the unbiased novel host environment backcrossed pathogens with genomes with a higher percentage from a single *Microbotryum* species (i.e. homospecific backcross) should perform better than offspring with a more mosaic genome (heterospecific backcrosses), but this study did not provide evidence for such an effect. The lack of evidence for negative epistatic interactions in these backcrosses may be reasonable considering the very small genetic distance between *M. silenes-dioicae* and *M. lychnidis-dioicae*[21,44] even though these two fungal species show reduced hybrid fitness in the form of sterility [32].

In addition, results obtained on the novel host environment suggest that greater genetic contribution from the *M. lychnidis-dioicae* species provided an infection advantage. The direction of backcrossing toward *M. lychnidis-dioicae* was higher in both of the mating-type treatments, where the most successful infection were mating type heterospecific, and significantly higher in one case than the 0.5 neutral expectation. A greater infection potential of *M. lychnidis-dioicae* than other *Microbotryum* species has been previously observed [21,44]. Thus, regarding post-mating isolation in *Microbotryum*, it is important that species-specific characteristics be considered in addition to the classification of intrinsic and extrinsic factors, and such an effect may also have contributed to higher infection rates by *M. lychnidis-dioicae*-backcrossed pathogens on the *S. latifolia* host.

### Potential for hybridization and backcrossing in *Microbotryum*

While a large number of studies utilize molecular tools for the analysis of present and past hybridization, the current study takes a different approach to illuminate the potential impact of inter-specific mating through controlled backcrossing experiments. F1-hybrids and backcrosses between the two closely related *Microbotryum* species *M. lychnidis-dioicae* and *M. silenes-dioicae* are highly viable on a natural host and a novel host, which supports the idea that hybridization and introgression have the potential to impact natural *Microbotryum* populations. There are several examples in plants and animals where hybridization seems to facilitate new evolutionary lineages [4,6,45], and in fungi hybrid speciation events also have been described [1,46,47]. The current study provides insights into the potentials for hybrid speciation in *Microbotryum* and for introgression via backcrossing of alleles from one species to another, which have both been suggested by molecular analysis of natural *Microbotryum* populations [24,44].

Reproductive isolation from the parental species is essential to the emergence of a new hybrid species. This can be achieved by changes in ecology or genetics (i.e. ploidy) that favor the production of offspring between hybrid genotypes [4,48,49]. The preference by F1 *Microbotryum* hybrids for conjugating with compatible mating type alleles from the same parental species may instead favor backcrossing over hybrid selfing, because F1-hybrid selfing is necessarily heterospecific at the mating type while backcrossing can be favored as homospecific. However, it should be noted that this would only be the potential influence of the mating type upon the process of backcrossing, which may not be strong enough to counter-programmed effects upon development that favor selfing in this organism.

## Conclusions

The present study reveals information about assortative mating that may disfavor hybridization as well as the potential dynamics of mating between hybrids and their parental species. The experimental approach of combining species-specific mating type alleles with competitive backcrossing treatments is a reasonable way to study pre-mating and post-mating reproductive isolation. In particular, the *Microbotryum* species differences that were difficult to resolve in previous studies may be revealed by the homogenizing influence of hybridization upon genome composition. Moreover, infection ability of backcrossed hybrids emphasizes the potential influence of hybridization upon the evolutionary genetics of this system.

## Methods

### Species and sampling

The sibling species *Microbotryum lychnidis-dioicae* and *M. silenes-dioicae* belong to the group of the anther smut-fungi on the Caryophyllaceae that are the cause of a sexually transmitted disease in which the host plant is sterilized by the replacement of pollen by diploid fungal spores [19,50]. During pollination the fungal spores, also called teliospores, are transmitted to other plants, where they germinate and undergo meiosis. Haploids propagate via yeast-like budding and conjugation occurs between cells of opposite mating types. The resulting dikaryotic hyphae enters the host and infects it systematically [26,51,52]. Teliospores of the parental species were collected from natural diseased populations of the hosts *S. latifolia* and *S. dioica* (Table 3) and the pathogen species were confirmed by sequencing of the elongation factor 1 (as in [21]).

**Table 3:** **Host species, geographic origin and year of isolation for the *Microbotryum *inoculum used in the current study**

**ID**	**Host species**	**Geographic origin**	**Year of collection**
Lam	*S. latifolia*	Lamole, Italy	2000
Ger	*S. latifolia*	Darmstadt, Germany	2000
Or	*S. latifolia*	Orsay, France	2000/2001
Sui	*S. dioica*	Olivone, Switzerland	2001
Fr	*S. dioica*	La Grave, France	2002
VdP	*S. dioica*	Valle de Pesio, Italy	2003

The two host species, *S. latifolia* and *S. dioica*, are perennial, dioecious species adapted to slightly different ecological niches but frequently co-occurring throughout much of their native European distribution [21]. Seeds of *S. latifolia* were obtained from a naturalized population in Belchertown, Massachusetts, for experimental inoculation studies. Also, seeds of the host *Silene colorata* were used as novel (or naive) host (http://www.tmseeds.com/product/1700/Salvia_Seeds), to which neither M-Sl and M-Sd is expected to exhibit adaptation [28]. *S. colorata* is an annual species, which does not harbor *Microbotryum* in nature [53]. Moreover, *S. colorata* constitutes a neutral host environment as it is phylogenetically equidistant from either *S. latifolia* or *S. dioica* which are themselves sister species [54].

### Production of F1-hybrid pathogens and their meiotic products

As this study employs *Microbotryum* hybrids in backcrossing experiments, it was first necessary to produce F1 genotypes by inoculating host plants with experimental crosses. With regard to the mating type locus, two types of *Microbotryum* hybrids were produced, depending on the direction of the cross: F1-hybrids with a1 mating type from M-Sl and a2 from M-Sd (referred to as M-Sl_a1_ × M-Sd_a2_) or F1-hybrids with a1 from M-Sd and a2 from M-Sl (referred to as M-Sd_a1_ × M-Sl_a2_). For clarity of presentation, haploid meiotic products obtained from the F1-hybrids are named according to their mating type, the origin of their mating type chromosome, and superscript "H" to indicate their hybrid origin. For example, an a1 mating type meiotic product originating from the F1-hybrid M-Sl_a1_ × M-Sd_a2_ would be named M-Sl_a1_^H^, and the a2 mating type product is M-Sd_a2_^H^ (Additional file 2).

Germination of the field-collected non-hybrid M-Sl and M-Sd teliospores was performed on potato dextrose agar (Difco), and following meiosis the resulting haploid sporidia were isolated from meiotic tetrads by micromanipulation (as in [55]). With this method, all four haploids derived from one single teliospore could be obtained, and thus, artificial selection upon haploid growth was not imposed by this approach. Mating types of the haploid cultures were determined by PCR-amplification of the pheromone receptor gene (as in [44]). To produce F1-hybrids, sporidia from M-Sl and M-Sd of opposite mating types were combined and used to infect *S. latifolia* by following the inoculation procedure of Hood et al. [56]. Isolates from different populations served as replicates for a given genotype; all crossings are listed in Table 4.

**Table 4:** Codes and origins for the haploid isolates produced from the F1-hybrids used in the pre-mating and post-mating experiment

***Isolate numbers***		Genotype of haploids*	**Genotype of F1-hybrids (diploids)**	**Origin of isolates**
***pre-mating***	***post-mating***			
1,2,3,4,5	1,2,3,4	M-Sl_a1_^H^	M-Sl_a1_ × M-Sd_a2_	Lam × Sui
6,7		M-Sl_a1_^H^	M-Sl_a1_ × M-Sd_a2_	Ger × VdP
8,9,10,11	8,9,10,11	M-Sd_a2_^H^	M-Sl_a1_ × M-Sd_a2_	Lam × Sui
12,13,14	12,13	M-Sd_a1_^H^	M-Sd_a1_ × M-Sl_a2_	Sui × Or
15,16	15,16	M-Sd_a1_^H^	M-Sd_a1_ × M-Sl_a2_	Fr × Lam
17,18		M-Sd_a1_^H^	M-Sd_a1_ × M-Sl_a2_	Fr × Or
19,20,21	19,20	M-Sl_a2_^H^	M-Sd_a1_ × M-Sl_a2_	Sui × Or
22,23	22,23	M-Sl_a2_^H^	M-Sd_a1_ × M-Sl_a2_	Fr × Lam
24,25		M-Sl_a2_^H^	M-Sd_a1_ × M-Sl_a2_	Fr × Or

*M-Sl_a1_^H^: haploid hybrid genotype with a1 mating type chromosome originating from *M. lychnidis-dioicae;* M-Sd_a2_^H^: haploid hybrid genotype with a2 mating type chromosome originating from *M. silenes-dioicae (*and vice versa for M-Sd_a1_^H^ and M-Sl_a2_^H^).

From plants that flowered with F1-hybrid pathogens, the diploid spores from smut-filled anthers were sampled and subjected to the same *in vitro* procedures for isolating haploid meiotic tetrads by micromanipulation as described above. Altogether, 25 hybrid meiotic isolates derived from crosses M-Sl_a1_ × M-Sd_a2_ and M-Sd_a1_ × M-Sl_a2_ were obtained (Table 4). All haploid cultures were maintained on potato dextrose agar and stored frozen in silica powder.

### Assortative mating in F1-hybrids

Haploid cultures were suspended in sterile deionized water and the sporidial concentration determined by using a Neubauer hemocytometer. Concentration were adjusted to contain 5.8 × 10^8^ cells/ ml. Crosses were performed by mixing 10 μl culture suspension of each mating type on 1.5% water agar (Bacto-Agar, 0.01% alpha-tocopherol solution (v/v)). The cells in the mixtures were incubated for 12 h at 15°C, at which time the cultures were fixed with 50 μl water containing 10% lactophenol blue solution (Sigma). For each cross the number of cells across 3 sections of the hemacytometer was recorded. Pairs of cells were counted as mated if the conjugation tube could be seen clearly under the microscope at 400 × magnification. To control for the viability of hybrid gametes, conjugation was assessed between haploids of opposite mating type derived from the same F1-hybrid.

For each of the conjugation mixtures the ratio of cells involved in conjugation over the total number of cells was computed. Whether there was a bias of the backcrossed mating towards having both mating type alleles derived from the same species (i.e. homospecific versus heterospecific mating) was assessed by a paired T-test with a significance level α = 0.05. To control for the viability of hybrid gametes, conjugation was assessed between haploids of opposite mating type derived from the same F1-hybrid (Additional file 1, C1-C8).

### Sources of post-mating isolation

#### Backcrossing and plant infection

To investigate extrinsic and intrinsic factors contributing to post-mating reproductive isolation, a backcrossing experiment was designed to manipulate the proportion of the F2-hybrid pathogen genome derived from M-Sl or M-Sd. For example, by backcrossing hybrid-produced gametes to non-hybrid M-Sl gametes, the F2-generation should contain 75% (on average) of autosomal alleles from M-Sl, whereas backcrossing to non-hybrid M-Sd gametes would contain 25% of autosomal alleles from M-Sl (Additional file 2). Here, the expectation is that a M-Sl-biased genome may achieve greater success, relative to a M-Sd-biased genome-, at infecting the native host *S. latifolia,* while in contrast there is no such expected infection bias on a novel host *S. colorata*.

The potential for intrinsic factors to impact reproductive isolation was investigated with regard to the contribution of the mating type loci in backcrossing. In *M. lychnidis-dioicae*, the region of suppressed recombination linked to mating type is quite large, comprising about 10% of the genome [55,57]. When backcrossing reunites the mating type alleles of one species (homospecific backcross), more of that species genome would be represented in the F2-hybrid compared to the heterospecific backcross. To evaluate the importance of intrinsic factors linked to mating type, the fitness of genomes with homospecific versus heterospecific backcrossing were compared on the novel host *S. colorata*.

Seeds of the host species *S. latifolia* and *S. colorata* were surface-sterilized in a solution containing 10% bleach, 50% ethanol and 40% sterile water and germinated at 24°C on 0.8% agar with 0.1 × MS salts [58]. A backcrossing 'competition' experiment was performed with 16 of the F1-hybrid isolates used in the pre-mating experiment (four per hybrid gamete genotype M-Sl_a1_^H^, M-Sl_a2_^H^, M-Sd_a1_^H^ and M-Sd_a2_^H^) (Table 4). For each of the 16 isolates, an inoculum was prepared by mixing sporidia of the hybrid-produced gamete with sporidia of the opposite mating type from both parental, non-hybrid cultures. This experimental setting reproduces the conditions under which there is a possibility for mate choice, with both homospecific and heterospecific conjugation being possible. To avoid possible pre-mating effects, mating preferences that were measured in the pre-mating experiment above were controlled for by adjusting cell concentrations (Additional file 1). Four μl of the resulting mixture were pipetted directly onto the apical shoot meristem on each host seedling according to the methods of Hood et al. [55]. For each inoculum (hybrid isolate plus parental cultures) 40 host plants were infected and in each plant, both homospecific and heterospecific backcross types had the chance to compete for infection of the host.

Treatments were randomly assigned to individual plants, which were randomized for position in the greenhouse. Six weeks after planting, plants started to flower and were scored as either diseased (fungal spores in the anthers detectable) or healthy. The diseased buds were sampled before opening to avoid secondary disease transmission to other plants.

#### Detection of backcross genotypes

Within each plant, the successful backcross infection was detected by genotyping of the mating type locus. DNA was extracted - using the Chelex (Bio-Rad) method for DNA extraction [59] from each flower filled with fungal spores. For the a1 mating type, species-specific PCR primers were designed with the online program WASP (http://bioinfo.biotec.or.th/WASP), based on Simple-Allele-Discriminating PCR protocol (SAP) [60]. The binding-site of the primer is within the sequence belonging to the STE-20 gene on the mating type chromosome [61]. PCR products were separated with electrophoresis and depending on which species-specific primers were used, the presence of a PCR product indicated the species genotype that won the competition. For the a2 mating type, SAP PCR was not possible, and thus sequencing was performed to determine which genotype was successful. For the a2 mating type, the locus 236, linked to the sex chromosome [61], was sequenced and ambiguous peaks on the chromatograms were analyzed to determine which a2 species was present. Due to difficulties in genotyping, only 522 of 999 infected plants were genotyped (Table 2). Primer sequence and PCR program are shown in Table 5. The number of homospecific and heterospecific backcrosses was quantified for each of the hybrid treatment and observations with the same hybrid genotype were pooled. The deviation from 0.5 ratio was checked by performing a two-tailed Binomial-Test.

**Table 5:** Characteristics of the PCR primers used in this study

**N**	**Loci**	**PCR primers (forward/ reverse)**	**Tm, °C**	**Specificity**
1	STE20-prA1	GTTCGATTCGGCAGCAT	58.5	a_1_ in M-Sl
		CACGACAGTCCAAGATTCAA		
2	STE20-prA1	CGCAGCTCTCACAAATGAGT	61.1	a_1_ in M-Sd
		ATCGTGGTAGCCCAACGATA		
3	236	GGAATCGACCATGCTAGTGG	60.0	a_2_ in M-Sl and M-Sd
		TAGTCGGAAGGTCGCTGAG		

## Competing interests

The authors declare that they have no competing interests.

## Authors’ contributions

All authors were involved in designing the experimental study. BB and EP completed the experiments. MED provided initial source of inoculum. BB drafted the manuscript with revisions by MEH, EP and DB. All authors read and approved the final manuscript.

## Supplementary Material

Additional file 1 **Complete list of sample size and observed conjugations of the pre-mating experiment.** Rates served as base for adjustments for the competition infection experiment. Click here for file

Additional file 2 **Figure showing the genetic composition of used crosses.** First row: Diploid parents (rectangles) and their meiotic products (circles) - haploid gametes that are crossed. Second row: Diploid F1-hybrids (rectangles) and their meiotic products (circles) that are backcrossed to parental haploids. Third row: Genotypes of the backcrossed hybrids (diploid). Click here for file
