# Supplementary material for: Experimental hybridization and backcrossing reveal forces of reproductive isolation in Microbotryum
Source: BMC Evol Biol. 2013 Oct 10;13:224. doi: 10.1186/1471-2148-13-224 (PMC3853205; doi:10.1186/1471-2148-13-224)
Supplement: Additional file 1 — Complete list of sample size and observed conjugations of the pre-mating experiment. Rates served as base for adjustments for the competition infection experiment. [file 1471-2148-13-224-S1.pdf]

Table S1. Sample size and observed conjugations of the pre-mating experiment. Rates served as base for adjustments for the 'competition' infection experiment.

Isolate no	Homospecific backcross N	con	prop	Heterospecific backcross N	con	prop	Proportions used for adjustment (µl)
1	95	14	0.29	120	6	0.10	25/ 75
2	101	18	0.36	106	11	0.21	21/ 36
3	82	17	0.41	111	10	0.18	18/ 42
4	105	3	0.06	117	5	0.09	131/ 88
5	204	39	0.38	201	94	0.47	-
6	201	32	0.32	201	40	0.20	-
7	205	64	0.62	205	128	0.62	-
8	88	4	0.10	105	18	0.34	82/ 22
9	106	11	0.21	95	7	0.15	36/ 51
10	102	15	0.29	123	5	0.08	26/ 92
11	111	15	0.27	110	9	0.16	28/ 146
12	102	35	0.69	101	19	0.38	11/ 20
13	99	21	0.42	0	0	0.00	18/ 19
14	203	73	0.72	202	66	0.33	-
15	113	7	0.12	108	6	0.11	61/ 68
16	103	13	0.25	106	7	0.13	30/ 57
17	281	93	0.66	610	294	0.32	-
18	274	95	0.69	452	185	0.41	-
19	109	17	0.31	96	14	0.29	24/ 26
20	113	16	0.28	119	12	0.20	26/ 37
21	336	106	0.63	317	146	0.46	-
22	108	7	0.13	121	6	0.10	58/ 76
23	108	8	0.15	101	8	0.16	51/ 47
24	571	165	0.58	610	292	0.48	-
25	315	92	0.58	317	140	0.44	-
C1	92	8	0.17	(Isolate: 1 x 8)			
C2	104	16	0.31	(Isolate: 2 x 9)			
C3	105	25	0.48	(Isolate: 3 x 10)			
C4	116	24	0.41	(Isolate: 4 x 11)			
C5	91	25	0.55	(Isolate: 12 x 19)			
C6	101	33	0.65	(Isolate: 13 x 20)			
C7	91	12	0.26	(Isolate: 15 x 22)			
C8	97	15	0.31	(Isolate: 16 x 23)			

N: observed number of cells per isolate; con: number of observed conjugations per isolate. Composition (in µl) of homospecific isolate/ heterospecific isolate used for inoculum of infection experiment. C1-C8: Control isolates that were tested for conjugation with sporidia from the same meiotic tetrad.
