# Supplementary figures and images for: Experimental hybridization and backcrossing reveal forces of reproductive isolation in Microbotryum
Source: BMC Evol Biol. 2013 Oct 10;13:224. doi: 10.1186/1471-2148-13-224 (PMC3853205; doi:10.1186/1471-2148-13-224)

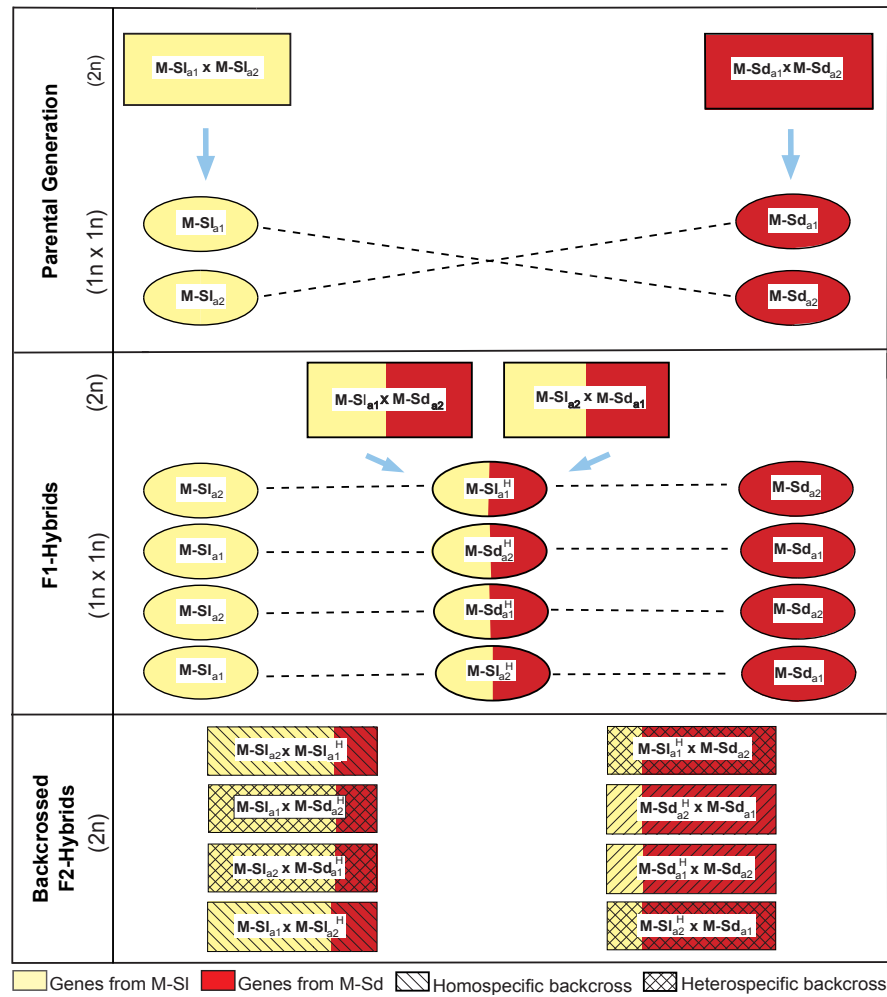

Supplement: Additional file 2 — Figure showing the genetic composition of used crosses. First row: Diploid parents (rectangles) and their meiotic products (circles) - haploid gametes that are crossed. Second row: Diploid F1-hybrids (rectangles) and their meiotic products (circles) that are backcrossed to parental haploids. Third row: Genotypes of the backcrossed hybrids (diploid). [file 1471-2148-13-224-S2.pdf]
